# Gastrointestinal Tract Morphometrics and Content of Commercial and Indigenous Chicken Breeds with Differing Ranging Profiles

**DOI:** 10.3390/ani11071881

**Published:** 2021-06-24

**Authors:** Joanna Marchewka, Patryk Sztandarski, Żaneta Zdanowska-Sąsiadek, Dobrochna Adamek-Urbańska, Krzysztof Damaziak, Franciszek Wojciechowski, Anja B. Riber, Stefan Gunnarsson

**Affiliations:** 1 Institute of Genetics and Animal Biotechnology, Polish Academy of Sciences, Jastrzębiec, 05-552 Magdalenka, Poland; p.Sztandarski@igbzpan.pl (P.S.); z.sasiadek@igbzpan.pl (Ż.Z.-S.); f.wojciechowski04@gmail.com (F.W.); 2 Department of Ichthyology and Biotechnology in Aquaculture, Institute of Animal Science, Warsaw University of Life Sciences, 02-786 Warsaw, Poland; Dobrochna_adamek@sggw.edu.pl; 3 Department of Animal Breeding, Faculty of Animal Breeding, Bioengineering and Conservation, Institute of Animal Science, Warsaw University of Life Sciences, 02-786 Warsaw, Poland; Krzysztof_damaziak@sggw.pl; 4 Department of Animal Science, Aarhus University, DK-8830 Aarhus, Tjele, Denmark; Anja.riber@anis.au.dk; 5 Department of Animal Environment and Health, Swedish University of Agricultural Sciences (SLU), S-532 23 Skara, Sweden; Stefan.Gunnarsson@slu.se

**Keywords:** range, broiler, ranging profile, animal welfare, gastrointestinal tract

## Abstract

**Simple Summary:**

Differences in the range use by poultry exist on the individual or breed level, even if equal opportunity of outdoor access is provided. Birds reared with access to the pasture consume some of the material found outdoors, such as plants, insects, and stones. The frequency of outdoor range use may be associated with the ingested material and the development of the bird gut. Optimal gastrointestinal tract morphometrics or small intestine microstructure are important for nutrient absorption and essential for poultry to resist diseases and assure welfare. Development of the gastrointestinal tract and its content could furthermore retrospectively indicate the birds’ ranging profile. The aim of the current study was to compare gastrointestinal tract morphometrics, small intestine microstructure, as well as the amount of pasture-originating material and feed ingested by the birds differing in their ranging profile, separately for the slow-growing broiler hybrid Sasso and green-legged partridge, a Polish indigenous breed of chicken. We found that the contents of the crop and gizzard of the moderate-indoor green-legged partridges were different from the indoor- and outdoor-preferring ranging profiles. In Sasso, the development of the villi in terms of their height and area in outdoor-preferring birds was different from that observed in other ranging profiles.

**Abstract:**

Optimal development of the gut is important for nutrient absorption and for poultry to resist diseases. The aim of the study was to compare gastrointestinal tract morphometrics, small intestine microstructure, as well as the amount of pasture matter and feed ingested by the birds with outdoor access presenting either an outdoor-preferring, moderate-outdoor or indoor-preferring ranging profile. Sixty non-beak trimmed birds per strain: broiler hybrid Sasso and Polish indigenous green-legged partridge were housed from week 5 to 10 in groups of 10, under conditions of EU organic meat chicken production. Pens with outdoor ranges were video recorded, to obtain frequencies of the birds’ range use. Statistical analysis was conducted applying generalized linear mixed models, applying the ranging profile as a fixed effect and pen as a random factor. The weight of the pasture matter in bird crops was the highest in moderate-outdoor profiled green-legged partridges, as compared to other ranging profiles (*p* = 0.04). In Sasso, villi in the small intestines were significantly higher in the outdoor-preferring compared to indoor-preferring profiled birds (*p* = 0.04), while their area was larger in the outdoor-preferring Sasso birds (*p* = 0.01). The level of development of the gastrointestinal tract and its content may be a potential indicator of the birds’ ranging profile and forage consumption.

## 1. Introduction

Usually, either slow-growing, indigenous or heritage chicken breeds are used for meat production in low-input systems. A slow-growing genotype may be defined as a bird with a daily weight gain below 45 g, attaining 2.2 to 2.5 kg in 56 to 81 days [1]. Similarly, McCrea et al. 2014 [2] defined the indigenous chicken breeds as ones that are derived from traditional lines, have a slow growth rate and can spend most of their life outdoors. Overall, slow-growing or indigenous chicken breeds are often characterized by being more active [3], robust and with excellent livability [1,4], due to a stronger immunological system [5].

Previous studies have shown that differences in the use of the range by domestic poultry exist on the individual, flock, or breed level, even if the equal opportunity of access to it is provided [6,7,8]. Birds less able to cope with the potential stress related to exposure to variable outdoor environmental conditions (i.e., sun, rain, snow, wind, storms), as compared to climate-controlled indoor housing [9,10], might be fearful of entering the range [11]. Therefore, Campbell et al. 2016 [11] profiled individual laying hens, differing in their ranging profiles, as outdoor-preferring, moderate-outdoor, and indoor-preferring. A study conducted based on the same birds, as in the current experiment, showed that a slow-growing chicken breed showed reduced welfare and outdoor range use levels, as compared to the indigenous one [12]. In addition, major differences between individuals for those parameters were identified within a breed [12]. We also showed that it is also possible to divide slow-growing and indigenous chickens into such ranging profiles [12].

Birds reared with access to the pasture, in addition to complete commercial diets usually fed ad libitum, will consume some quantities of the material found in the outdoor environment, such as fibrous plant materials, insects and grit stones. Feed represents a major component in the cost of poultry production, regardless of the production system. Slow-growing and indigenous chicken breeds in low-input systems are known for poorer productive performance, seen as a high feed conversion ratio [13], which increases the cost of production. Rearing birds with access to a range covered with vegetation might help to reduce feed intake cost not only as range forage consumption can be added to the chickens’ dietary intake [14,15,16] but also because it can enhance the utilization of the standard cereal-based diet [17,18]. Moreover, it is necessary that the dietary components are available to the birds’ body as a result of the forage digestion in the well-developed gastrointestinal tract and nutrients absorption from the healthy small intestine. However, large individual variation in forage consumption, ranging from 0 to 48% of the forage in the crop, was observed in pullets and broilers that allowed pasture access [19,20,21]. It remains unknown if associations exist between birds belonging to a particular ranging profile and their outdoor foraging levels.

Ingestion of pasture vegetation has been found to stimulate an improved development of the crop as an intermediate storage organ in free-range broilers [21]. To our knowledge, the gastrointestinal tract morphometrics—for instance, weights of the crop, proventriculus, gizzard, liver, duodenum, jejunum, and ileum, as well as lengths of duodenum, jejunum, and ileum—have previously been used for comparison of broilers with various genetic origins [22] as well as different ages [23], but no study on the effects of ranging behavior on the gastrointestinal tract morphometrics has been performed.

The small intestine is designed to allow for maximum absorption of dietary components. The healthy and functional gut is characterized by specific microstructure parameters. Long villi are associated with the increased total luminal absorptive area and subsequent satisfactory digestive enzyme action and higher transport of nutrients [24]. Shallow crypts reflect the prolonged survival of villi without the need for renewal [25,26]. The microscopic structure of the small intestine in terms of villus height and crypt depth is considered the main indicator of intestinal development, health, and functionality [23,27]. Intestinal absorption processes evolved differently between genetic lines, leading to wide differences in the efficient digestion of nutrients in diverse bird strains [28,29] but the alterations in the intestinal structure between birds differing in the ranging profiles were not investigated in any slow-growing or indigenous chicken breed.

The aim of the current study was to compare gastrointestinal tract morphometrics, small intestine microstructure, as well as the amount of pasture-originating material and feed ingested by the birds differing in their ranging profile, separately for the slow-growing broiler hybrid Sasso (Hendrix Genetics BV and Sasso) and green-legged partridge, a breed of chicken indigenous to Poland [30], due to the large difference between their body weight and body size. The average body weight of green-legged partridge roosters reaches around 2.5 kg and hens around 1.7 kg, which is achieved at about five months of age. In comparison, Sasso birds reach a slaughter weight of 2.3 to 2.9 kg at about two months of age. Nevertheless, both Sasso birds and green-legged partridges are well adapted to forage on the outdoor ranges, having high resistance to low temperatures and diseases, while the meat is characterized by a good taste and quality [31]. Furthermore, we examined the correlations between all collected measures for each of the two breeds.

## 2. Materials and Methods

The experiment was carried out in the Mazovian region in Poland in August to September of 2018, at the facilities of the experimental farm of the Institute of Genetics and Animal Biotechnology of the Polish Academy of Sciences.

### 2.1. Animals, Housing and Management

Sixty non-beak-trimmed, mixed-sex birds of each of two breeds (total n = 120 birds)—green-legged partridge and Sasso (for consistency, both Sasso and green-legged partridge will be referred to as “breed”, even though Sasso is a commercial hybrid)—were used in the experiment. Prior to week five of age, birds were reared according to the breeding company guidelines in the single breed groups of 300 birds, one group per barn, all located at the same experimental farm in a littered pen with perches. Feed and water were provided ad libitum, while the light was natural. The climate conditions were controlled automatically and infrared heating lamps were used. During this rearing period, birds were not allowed outdoor access. At the age of five weeks, 120 healthy birds, as assessed by the experimental facilities veterinarian, with a similar body weight within each breed (on average 2030.6 ± 68.9 g for Sasso and 705.9 ± 8.5 g for green-legged partridge) were selected and moved from their rearing facilities to the experimental house. The birds were randomly assigned to the mixed sex, single breed groups of 10 birds housed in 12 pens, where they were housed until 10 weeks of age. Mortality was monitored from week five, but no birds died during the experiment. The housing conditions exceeded EU requirements of organic meat-purpose chicken production [32]. Indoor pens were 2.5 m × 3.5 m large, resulting in a stocking density at slaughter age of 1.4 kg/m^2^ for green-legged partridge and 2.7 kg/m^2^ for Sasso. A layer of sawdust litter was added on top of the floor, while there was a 0.5 m stripe covered with sand next to the wall. The litter was renewed weekly and pens were partly cleaned daily, when necessary. In each pen, there were two 80-cm-long wooden, horizontal perches with two perching levels, one at the height of 15 cm and the second at 40 cm, respectively. The perching poles were 50- × 50-mm-thick and had rounded edges. Each pen had direct access to an individual outdoor range (3.5 m × 30 m) through a pop hole (45 cm high × 50 cm wide), providing 10.5 m^2^/chicken, thus considerably above the required 4 m^2^/chicken in the organic regulation. All the outdoor ranges had equal vegetation coverage regarding botanical composition at the start of the experiment and height but no trees or shelters were present. The grass was mowed one week before the onset of the experiment. Each range area was provided a half-automatic bell drinker and a wooden box (1 m × 1 m) filled with sand.

The birds were habituated for 48 h to the new housing and social situation before pop holes were opened daily from 7.00 until 19.00 h to allow for individual birds’ recognition; all birds were fitted with a small, laminated paper mark attached to the birds’ back by fitting two elastic bands around the wings. Ten different colors of the marks were randomly assigned in each pen to the individual birds. Birds were equipped with their color mark during the entire experiment, and they were inspected twice a day.

Commercial organic certified pelleted feed (EU Mastgeflügel Pellets ab 4 LW, Agro-Handel Mirsk, Mirsk, Poland) was used to nourish the birds. The feed was composed of wheat, maize, sunflower meal, pea, soybean meal, legumes mix, gruel corn, monocalcium phosphate, soybean oil, calcium carbonate and supplements [12]. The manufacturer did not disclose proportions of the components; however, the dietary composition of the feed was designed to meet slow-growing broilers’ nutritional requirements under the organic production circumstances at between 5 and 10 weeks of age. It contained 20% protein, 5% fat, 6% fiber, 6.5% ash, 1.05% calcium, 0.82% lysine, 0.65% phosphorus, 0.34% methionine and 0.16% sodium. No coccidiostats or other medications were used. Feed and water were available ad libitum. The feed contained 11.8 MJ metabolizable energy per kg. All the components in the feed may be used in organic production in accordance with EU regulations [32,33].

The birds were provided only natural light through uncovered windows as the room had no artificial lights. Light hours during the experimental period ranged from 12.7 h to 15.7 h/day, depending on the natural day length. There was natural ventilation in the building. Indoor climate parameters were automatically and continuously recorded by a measuring device (Davis Vantage Pro, Hayward, CA, USA). The temperature recorded in the building during the experiment ranged between 19 and 26 °C, while the humidity ranged from 47 to 71%. During the day, the outside temperature ranged between 12 and 28 °C, outside humidity ranged from 46 to 99% and wind speed from 0 to 24 m/s.

### 2.2. Observations of Ranging Behavior

For behavioral observations of birds, the 12 ranges were video recorded simultaneously and continuously using six cameras (DMIP2401IR-M-IV IP 4 Mpix, BCS company, Warszawa, Poland), each completely covering two range areas. The video recordings were automatically saved on the network recorder (BCS-NVR0401-IP 4 channel BC, BCS company, Warszawa, Poland), and from these the birds’ behaviors were analyzed by the same trained and experienced person, using the Chickitizer program [34]. The program is a computer application specially developed to record data about the location of animals in enclosed, predefined areas, as it enables graphic mapping of the experimental layout (distribution of compartments) with a single mouse click. From the recorded videos, three days were chosen per week of experiment (five weeks), selected to avoid the day on which welfare assessment took place. On each of those days, at three times of the day (morning—starting at 8:00, noon—starting at 13:00, and evening—starting at 18:00), a three-minute-period with 10 s sampling intervals was set and repeated after 10 min. The observer recorded each of the experimental birds’ absence as “0” or presence as “1” in the range. The possible frequency of outdoor use in the current study was between 0 and 1620. This results from the observation protocol where there were six samplings (one sampling/10 s, making up 1 min) ∗ 3 min ∗ two bouts ∗ three times of day ∗ three days each week ∗ five weeks = max. 1620.

### 2.3. Gastrointestinal Tract Measurements

At the end of the experiment, birds were weighed and culled by CO_2_ inhalation. The time of the culling was at noon to allow birds to use the pasture between daily pop-hole opening at 7 am for 4 h until the culling. Thereafter, the birds were transferred to a supine position and the thorax was opened by cutting the ribs and coracoid on the left side with scissors. The crop, the proventriculus and the gizzard were excised and weighed. The small intestine sections, comprising the duodenum (from the gizzard junction to the bile duct junction), the jejunum (from the duodenum end to the Meckel’s diverticulum), and the ileum (from the Meckel’s diverticulum to cecal junction) were stretched and measured, as was the caeca and colon.

### 2.4. Separation of the Crop, Proventriculus and Gizzard Contents

In the current study, we followed, with some modifications, the method of the separation of the crop, proventriculus and gizzard contents described previously in detail for laying hens [35]. Crop, proventriculus and gizzard were opened with a scalpel. The proventriculi of all birds were inspected; however, all were found empty of solid content. After opening the crop, it was turned inside out and the content was collected in a bowl. The remaining content was flushed from the inner surface with water. The crop was dried with a paper towel and reweighed to calculate the whole crop content. The crop content was then diluted with water to separate single components from each other. Contents of the crop were visually separated into fractions: feed, pasture, and feathers. The term ‘pasture matter’ comprises grass, as well as clover, grass seed and other plant particles. Weights of each content type were determined after drying with a paper towel to avoid too much water. The empty crop was weighed. In gizzards, the feed, pasture, and feather particles were crushed thoroughly by the grit composed of the gravel and stones. Thus, it was not possible to select the particles. The identified grit was not considered for the analysis; therefore, it was removed manually. The remaining content of the gizzard was divided into three fractions by passing it through three sieves (mesh width: fraction 1:500 to 1000 µm; fraction 2:1000 to 1500 µm and fraction 3:1500 to 2000 µm) by washing under running water. What remained on each sieve was collected. Thereafter, respective fractions were weighed, as was the empty gizzard. The crop and gizzard content was weighed using a laboratory weighing scale (Axis ATA220, Poland) with an enclosure and reading unit of 0.001 g.

### 2.5. Histological Measurements

**Figure 1:**
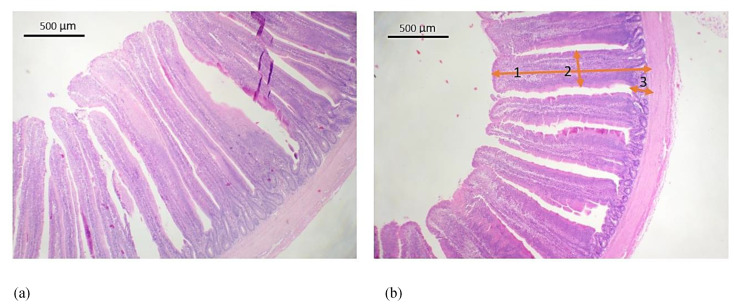
Small intestinal epithelium morphometrics: (**a**) green-legged partridge and (**b**) Sasso. The numbers indicate the morphometric measurements taken: 1—the villus height; 2—the villus width; 3—the crypt depth. Calculations using villus height and width at half height gave the villus surface area, H-E, scale bar 500 μm.

For the histological analysis, segments of about 1 cm in size were removed from the jejunum (2 cm anterior to Meckel’s diverticulum). The tissues were fixed in Bouin’s fluid, dehydrated, and subjected to the standard histological procedure. The paraffin-embedded tissues were cut transversely on a rotary microtome (Leica RM2265, Nussloch, Germany) into 6-μm-thick sections and stained with the hematoxylin-eosin (H-E) method. For the microscopic analysis of the preparations, a Delta Optical Pro microscope (Nowe Osiny, Poland) with a DLT-Cam PRO 5MP camera (Nowe Osiny, Poland) were used. Morphometric measurements were made using Delta Optical DLT-Cam Viewer software (Nowe, Osiny, Poland). The following measurements were taken: intestinal villi length, intestinal villi width and intestinal crypt depth (Figure 1). These parameters were measured for each individual on 50 well-aligned villi and corresponding crypts from each section of all intestinal segments and averaged for each bird. The heights of the villi were defined from their tip to the base and the widths were measured at the half height point. A standard method for villus:cript ratio determination was used, where the ratio of villus to crypt was estimated by dividing the villus height by the crypt depth in all measured villus and crypts [36]. The surface area of the villus was estimated by considering a villus as a cylindrical structure. Villus surface area was calculated using the formula: villus absorptive surface area = 2π × (average villus width/2) × villus height [36].

### 2.6. Statistical Analysis

Birds representing either of the breeds were divided into three ranging profiles using rank-frequency distribution (a discrete form of a quantile function in reverse order, giving the size of the element at a given rank) of their range use frequency summed over all the observation periods—i.e., between 0 and 1620 times. All the birds within a breed were assigned a rank based on their individual frequency of outdoor use. We segmented the rank distribution of the birds into three ranges: outdoor-preferring ranging profile, with the mean value of 506.1 ± 47.9 outdoor uses per bird during all observation periods in Sasso (n = 14) and 502.6 ± 22.5 for green-legged partridge (n = 24); moderate-outdoor ranging profile, with the mean value of 219.6 ± 18.8 outdoor uses per bird during all observation periods for Sasso (n = 19) and 332.4 ± 13 outdoor uses per bird for green-legged partridge (n = 21); and indoor preferring ranging profile, with the mean value of 89.8 ± 11.7 outdoor uses per bird for during all observation periods Sasso (n = 27) and 223.9 ± 12.1 outdoor uses per bird for green-legged partridge (n = 15). The rank intervals were equal; however, the number of birds in each group was not equal (modified from Campbell et al., 2016). By using this method, we overcame the issue of some birds having the same frequency of outdoor uses, but divided into various ranging profiles if the ringing profiles would be created based on equal bird numbers per ranging profile group.

Statistical analyses were performed with SAS 9.4 (SAS Institute, Inc., Cary, NC, USA). The GLIMMIX procedure was used to perform generalized linear mixed models for the gastrointestinal tract measurements using either normal or gamma distribution where appropriate, applying the ranging profile group as fixed effects in the model. Pen was included in the model as a random effect. The assumptions of homogeneity of variance and normally distributed residuals were examined visually using the conditional Studentized residuals plots. The results are shown as means with corresponding standard errors, and *p*-values below 0.05 were considered significant, while between 0.05 and 0.06 were considered a statistical significance trend. Tukey’s post hoc test was performed to investigate significant differences between test groups. Spearman correlations, calculated using the PROC CORR script, were used to test the relationships for all the gastrointestinal tract measurements and body weights separately for green-legged partridges and Sasso birds.

## 3. Results

### 3.1. Ranging Profile Effect on Gastrointestinal Tract Morphometrics and Content of Green-Legged Partridges

The effect of ranging profile was found on the weight of the pasture matter identified in the crop of green-legged partridges (Table 1). The weight of the pasture matter was the highest in moderate-outdoor ranging profiled birds, as compared to two other ranging profiles (*p* = 0.0451; Table 1). Moreover, there was a trend (*p* = 0.0517) for a significant difference between weight of the second fraction (1000–1400 µm) in the gizzard between ranging profiles. The highest weight of this fraction was identified in the indoor-preferring birds, as compared to two other ranging profiles. No significant differences were found regarding villi height or area between green-legged partridges with different ranging patterns.

### 3.2. Ranging Profile Effect on Gastrointestinal Tract Morphometrics and Content of Sasso

The effect of ranging profile was found on selected histological measurements (Table 2). The villi were on average significantly higher in the outdoor-preferring ranging profiled birds, as compared to indoor-preferring ones (*p* = 0.0393; Table 2). Moreover, the villus area differed significantly between all ranging profile groups (*p* = 0.0126; Table 2), being the highest in the outdoor-preferring birds and the smallest in the indoor-preferring ones, while moderate-outdoor ranging birds had an intermediate size of the villus area.

### 3.3. Correlations among Gastrointestinal Tract Morphometrics/Body Weight of Green-Legged Partridges

Correlations were calculated for gastrointestinal tract morphometrics and body weight for green-legged partridge birds (Table 3). Positive correlations were identified between crypt depth and the weight of pasture matter identified in the crop and the lengths of the small intestine with the weight of the empty crop. Furthermore, the weight of the empty gizzard was positively correlated with the small intestine, caeca, and colon lengths, as well with villi widths. The weight of the full gizzard was positively correlated with small intestine lengths and villi widths, while villi widths with the weight of the fraction 3 (1400–1800 µm) were found in the gizzard.

Moreover, significant correlations were identified between the metrics within the same gastrointestinal tract sections, as presented in the Table 3. The weight of the full crop was highly and positively correlated with the feed content. The empty gizzard was positively correlated with the full gizzard and fraction 1 weight, while the full gizzard weight was positively correlated with the weight of fraction 1 and 2. The length of the small intestine was positively correlated with the caeca and colon lengths. The villus area was positively correlated with the villus height and width. The villus height ratio to crypt depth was positively correlated with the villus height, crypt depths and villus area.

### 3.4. Correlations among Gastrointestinal Tract Morphometrics/Body Weight of Sasso

Correlations were identified among gastrointestinal tract morphometrics and body weight for Sasso birds, as presented in Table 4. Positive correlations were identified between the weight of the pasture matter in the crop with the lengths of the small intestine and villus height to crypt depth. Villus area was positively correlated with the weight of the fraction 3 (1400–1800 µm) found in the gizzard. Villi width was negatively correlated with the colon lengths.

The weight of empty crop was positively correlated with the weight of full crop but also with the weights of the feed content (Table 4). Pasture matter weight was positively correlated with the weights of the full crop and feed content, and the feed content weight with the full crop weight. The empty gizzard was positively correlated with the weights of the full gizzard and all three fractions, as was the full gizzard. The weight of fraction 1 was positively correlated with fraction 2, and the weight of fraction 2 with fraction 3. As for green-legged partridges, the length of the small intestine was positively correlated with the caeca and colon lengths. The villus area and villus height ratio to crypt depth were positively correlated with villi height, width, and crypt depth.

## 4. Discussion

In each of the breeds, the ranging profile had an effect on different parameters. In green-legged partridges, the ranging profile affected pasture matter weight identified in the crop and fraction of the particles of the size between 1000 and 1400 µm identified in the gizzard. The crop and gizzard are very closely linked parts of the gastrointestinal tract in poultry. The capacity of the crop is closely related to the capacity of the gizzard since the crop supplies the feed to the gizzard in successive quantities, as required [37]. According to Heuser [38], there is always feed in the gizzard when there is feed in the crop, suggesting that a more or less empty gizzard will cause the feed to pass directly through the crop to the gizzard.

Even though some hydrolysis of starch occurs in the crop [39], the main function of the crop is to store and soften ingested food before it is transported to the proventriculus and the gizzard [40]. The capacity of storage seems to increase with increased intake of feed. Previous studies have shown, based on the examinations of the performance, muscle fiber structure and feed consumption, that the efficiency of pasture use is closely linked to the genetic background of the birds [41,42]. Although the statistical comparison of the breeds was not performed in the current study, the weight of the pasture matter in the crop was three times higher in moderate-outgoing green-legged partridge birds, as compared to moderate-outgoing Sasso. This difference between investigated breeds becomes even larger in relation to their body weights. For Sasso, in contrast to green-legged partridge birds, the weight of the pasture matter in the crop did not significantly differ between ranging profiles. However, a significant positive correlation was identified between empty and full crop weight only for Sasso.

Moreover, the composition of the diet can significantly affect the feed passage rate [43]. It has been found that grass biomass in the crop represented between 2.5 and 4.5% of the total feed intake in grazing range birds [21]. In the current study, we found a comparable result to this value of the pasture biomass in the crop of moderate-outdoor green-legged partridges, representing on average 2.7% of their total crop content.

In the crops of the moderate-indoor green-legged partridges, there was significantly more pasture matter identified, as compared to other ranging profiled birds of that breed. There was, however, the same amount of feed identified in the crop of green-legged partridges from all ranging profiles. In broiler chickens of a slow-growing genotype, birds’ performance primarily depended on the intake of the cereal-based feed [21], which in the current study was located indoors. The role of the pasture intake in broilers with range access remains unclear. On the one hand, increased pasture intake in broilers improved the consumption of the cereal-based feed, even though the levels of forage intake were low [17]. In contradiction, another study showed that the ingestion of plant particles by domestic poultry reduced their intake of compound feed without affecting performance, reducing feed costs [18]. Moreover, the intake of roughage from an open-air run depends on the motivation of the birds to forage [44]. The amount of pasture in the crop of green-legged partridges could have a behavioral background. In hens, the moderate-indoor birds clearly contrasted in their behavior with the outdoor-preferring or indoor-preferring hens as they had inconsistent range access patterns [11]. Coping styles are strongly associated with the levels of stress vulnerability, including the individual vulnerability to disease [45]. An understanding of the ranging behavior of birds in relation to pasture vegetation ingestion is important not only for realizing the full potential of range access but also to ensure the health and welfare of all the birds in the flock regardless of the ranging profile. Therefore, further investigations should focus on the behavioral background of individual birds in relation to foraging, considering the indoor situation and social context of those birds.

Fibrous material, as is the pasture matter, increases the size, and in turn, the empty weight of the gizzard, resulting in a greater capacity of this organ, which in turn may increase the flow of feed through this organ [46]. Hence, we have identified a strong significant positive correlation between the weight of the empty and full gizzard in both breeds. Nevertheless, differences in the size of the empty gizzard between ranging profiled green-legged partridges were not detected, nor were they detected in Sasso birds. We found, however, that the fraction of particles of intermediate size, between 1000 and 1400 µm, identified in the gizzard of green-legged partridges showed a tendency to be higher in the indoor-preferring ranging profile, as compared to moderate-indoor and outdoor-preferring birds. This may indicate that the gizzards of the indoor preferring birds were not as active as in other ranging profiled birds of that breed, cumulating the intermediate fraction of the feed. Moreover, the weight of the full gizzard was positively correlated in green-legged partridges with the finest and intermediate size fractions, while in Sasso it was positively correlated with all the fractions. This may indicate lower activity and passage of the digested matter of larger size through the gizzard in Sasso birds.

Variable diet provided by the outdoor environment has profound effects on the development of all digestive tract organs in poultry [47]. The capacity of the digestive tract has been found to depend on the age, where the effect of the types of feed on the content in jejunum, ileum and caeca was observed when hens were 53 weeks old [48], while not as early as the age of the birds in the current study. This may be the reason why we did not observe any differences in small intestine, caeca, and colon lengths between ranging profiles in either of the breeds in the current study. However, regardless of the ranging profile in Sasso, the pasture matter weight in their crop was positively correlated with small intestine length, suggesting a faster speed of growth of Sasso birds, as a result of more intensive genetic selection of this breed, as compared to green-legged partridges.

The stable development of the gastrointestinal tract was confirmed by the positive correlations between the weight of the empty crop and gizzard with the small intestine overall length, and empty gizzard with caeca and colon lengths only for green-legged partridges. This was not observed in Sasso birds, since this breed underwent more intensive genetic selection as compared to green-legged partridges. A significant side effect of the intensive genetic selection of high-performance meat production poultry lines has been a disruption in the balance between the relative slow growth of their organs and their extremely rapid increase in muscle mass [49,50].

In Sasso birds, the ranging profiles differed with regard to the histological measurements in the small intestine, i.e., villi height and villus area. Both measures were the lowest for indoor-preferring birds, as compared to outdoor-preferring ones. The villus area was also smaller in moderate-indoor birds compared to outdoor-preferring ones. Intestinal villi and absorptive epithelial cells play significant roles in the final phase of nutrient digestion and assimilation [51]. Therefore, the indoor-preferring Sasso birds could be characterized by having the poorest digestion and assimilation potential. Fibrous material from the range increases the quantity of non-starch polysaccharides (NSP) in the gastrointestinal tract [52]. Insoluble NSP can have beneficial effects in the gastrointestinal tract, such as increasing the weight and size of the gizzard, pancreas, and liver, as well as increasing the intestinal villus height and subsequently the surface area [53,54]. It was suggested that the intestinal adaptations in meat-type chickens, as the ones observed in the outdoor-preferring birds having access to the roughage from the pasture, may be an attempt to compensate for the low functionality of their gastric area [22]. However, the causal relationships between the ranging behaviour, pasture ingestion and the development of the small intestine morphological characteristics remain unclear. Most importantly, the causal relationship behind the clearly poorer development of the intestinal villus height and subsequently their surface area in the indoor-preferring Sasso remains uncertain. Moreover, a negative correlation between villus width and colon lengths was identified for Sasso only. It has previously been found that the colon possesses very high absorptive capacity in chickens [55]. However, it remains unclear if the visceral organs adaptation revealed by colon length can be viewed as an attempt to compensate for the low functionality of the histological development of the intestine or the histological modifications such as the width of the villi or the gastric area. Further investigations should unfold whether the indoor preference reduces the forage intake, resulting in the poor development of the intestinal microstructure, or whether birds with suboptimal small intestine development prefer to stay indoors due to increased fearfulness and negative emotional states.

Several studies in chickens have reported that epithelial cell turnover and nutrient absorption were found to be correlated with the villus height to crypt depth ratio [56,57]. De Verdal et al. 2010 [22] identified the ratio between 7.73 in duodenum to 4.94 μm:μm in ileum, indicating a higher rate of epithelium turnover in the proximal part of the small intestine. In the current study, we found the ratio to be in green-legged partridges from 5.3 ± 0.2 in indoor-preferring birds and 5.4 ± 0.3 in moderate-indoor to 6.2 ± 0.3 in outdoor-preferring birds, while in Sasso it ranged from 6.2 ± 0.3 in outdoor-preferring birds to 6.6 ± 0.3 in moderate-indoor birds. Only in Sasso was the pasture matter weight in the crop positively correlated with the villus height to crypt depth, which has an unknown underlying mechanism.

In the current study, only for green-legged partridges was the villus width positively correlated with the empty and full gizzard weight, as well as with the fraction of the largest particles identified in the gizzard. Yamauchi [58] hypothesized that stimulation of the intestine absorptive function results in an adaptive compensatory enlargement of the villi. This is also in agreement with the findings that the villus width was higher in birds selected for lower digestive efficiency [22].

## 5. Conclusions

In conclusion, in Sasso birds, we detected that the development of the villi height and area in outdoor-preferring birds was different from that observed in birds with other ranging profiles and therefore they had the best digestion. Furthermore, we found that the contents of the crop and gizzard of the moderate-indoor green-legged partridge birds were different from birds with the indoor- and outdoor-preferring ranging profiles, which may depend on a different feeding pattern. Moreover, we identified that for each of the investigated breeds the correlations between the gastrointestinal tract and its content measurements were different. This difference probably depends on the degree of genetic selection each strain has been subjected to, also resulting in different growth rates. Based on the current results, we suggest that the different gastrointestinal tract morphometrics and content parameters in commercial and indigenous chicken breeds may indicate differences in the birds’ ranging profiles and forage consumption patterns during the production cycle.

## Figures and Tables

**Table 1:** The gastrointestinal tract morphometrics and content of green-legged partridge chickens with differing ranging profiles, presented as mean ± SEM and its associated test statistics.

Variable		Ranging Profile of Green-Legged Partridge (n = 60)			Pooled SEM	F Value	*p* Value
		Outdoor-Preferring (n = 24)	Moderate-Indoor (n = 21)	Indoor-Preferring (n = 15)			
		Mean					
Body weight (kg)		1.2	1.1	1.1	0.1	1.53	0.2322
Crop (g)							
	Empty	4.9	5.0	4.6	0.4	0.22	0.8074
	Full	10.3	13.3	9.4	2.2	0.81	0.4536
	Feed	4.7	7.9	3.7	1.9	1.08	0.3533
	Pasture matter	0.05 ^B^	0.3 ^A^	0.03 ^B^	0.05	6.26	0.0451
	Feather	0.07	0.1	0.02	0.03	0.81	0.4796
Gizzard (muscular stomach) (g)							
	Empty	34.5	31.7	31.8	1.9	0.59	0.5604
	Full	53.1	48.4	53.5	2.4	0.74	0.4862
	Fraction 1 (600–1000 µm)	8.2	6.9	7.3	1.1	0.39	0.6794
	Fraction 2 (1000–1400 µm)	7.6 ^B^	7.5 ^B^	11.2 ^A^	1.1	3.06	0.0517
	Fraction 3 (1400–1800 µm)	1.7	2.1	2.4	0.3	1.81	0.1817
Digestive tract measurements (cm)							
	Small intestine length (duodenum + jejunum + ileum)	140.1	131.4	138.4	5.2	0.74	0.4858
	Caeca length	33.9	34.3	33.5	1.1	0.10	0.9087
	Colon length	11.8	11.6	10.8	0.3	2.53	0.0960
Histological measurements (μm)							
	Villi height	1445.2	1558.2	1522.8	70.2	0.74	0.4862
	Villi width	165.4	158.3	161.7	8.0	0.22	0.8036
	Crypt depth	236.1	239.4	239.6	10.1	0.04	0.9630
	Villus area (µm^2^)	236,713.9	247,802.4	247,540.9	14,745.4	0.06	0.9378
	Villus height/crypt depth	6.2	6.6	6.4	0.3	0.35	0.7098

Means in the same row indicated by the different superscript letters (A, B) differ significantly (*p* < 0.05).

**Table 2:** The gastrointestinal tract morphometrics and content of Sasso chickens with differing ranging profiles, presented as mean ± SEM and its associated test statistics.

Variable		Ranging Profile of Sasso (n = 60)			Pooled SEM	F Value	*p* Value
		Outdoor-Preferring (n = 14)	Moderate-Indoor (n = 19)	Indoor-Preferring (n = 27)			
		Mean					
Body weight (kg)		3.0	2.8	3.1	0.5	0.82	0.4517
Crop (g)							
	Empty	9.5	8.0	9.3	0.7	1.33	0.2830
	Full	32.8	20.2	39.1	6.5	1.80	0.1852
	Feed	22.1	11.9	27.6	5.4	1.52	0.2378
	Pasture matter	0.1	0.1	0.2	0.07	0.82	0.4524
	Feather	0.01	0.01	0.02	0.008	0.57	0.6745
Gizzard (muscular stomach) (g)							
	Empty	49.3	42.8	49.5	4.2	0.84	0.4433
	Full	67.9	64.7	74.5	8.5	0.33	0.7186
	Fraction 1 (600–1000 µm)	9.6	12.2	12.3	2.7	0.13	0.8785
	Fraction 2 (1000–1400 µm)	2.8	4.7	5.1	1.2	0.75	0.4838
	Fraction 3 (1400–1800 µm)	2.3	2.1	2.4	0.7	0.06	0.9464
Digestive tract measurements (cm)							
	Small intestine length (duodenum + jejunum + ileum)	223.6	216.7	231.2	12.3	0.49	0.6161
	Caeca length	47.4	49.1	48.6	2.1	0.19	0.8308
	Colon length	14.4	13.8	14.7	0.6	0.55	0.5839
Histological measurements (μm)							
	Villi height (µm)	1204.3 ^A^	1153.7 ^AB^	1038.7 ^B^	45.2	3.60	0.0393
	Villi width (µm)	158.1	146.9	148.6	4.9	1.47	0.2456
	Crypt depth (µm)	198.8	216.3	199	7.1	1.97	0.1565
	Villus area (µm^2^)	188,655.9 ^A^	169,771.2 ^B^	154,128.2 ^C^	7237.1	5.05	0.0126
	Villus height/crypt depth	6.2	5.4	5.3	0.3	2.65	0.0866

Means in the same row indicated by the different superscript letters (A, B and C) differ significantly (*p* < 0.05).

**Table 3:** Spearman correlations between gastrointestinal tract morphometrics and content of green-legged partridge chickens.

	Crop (g)				Gizzard (Muscular Stomach) (g)					Digestive Tract Measurements (cm)			Small Intestine Histological Measurements				
	Full	Feed Content	Pasture Matter	Feather	Empty	Full	Fraction 1 (600–1000 µm)	Fraction 2 (1000–1400 µm)	Fraction 3 (1400–1800 µm)	Small Intestine Length (Duodenum + Jejunum + Ileum)	Caeca Length	Colon Length	Villus Height (µm)	Villus Width (µm)	Crypt Depth (µm)	Villus Area (µm^2^)	VILLUS Height/Crypt Depth
Crop (g)																	
Empty	0.23	0.09	0.06	0.16	0.32	0.25	0.17	0.07	−0.07	0.47 **	0.03	0.27	0.07	−0.20	0.07	−0.07	0.01
Full		0.96 ****	0.19	0.10	−0.02	0.11	0.15	0.16	0.08	0.15	0.03	0.24	−0.08	0.00	0.21	−0.06	−0.18
Feed content			0.14	0.06	−0.04	0.11	0.15	0.19	0.09	0.11	0.09	0.25	−0.09	−0.01	0.15	−0.08	−0.15
Pasture matter				−0.07	0.00	−0.15	0.13	−0.24	−0.03	0.04	−0.18	0.24	0.07	−0.15	0.58 ***	−0.03	−0.27
Feather					−0.03	0.05	0.01	0.12	−0.10	0.07	−0.13	−0.03	−0.22	−0.19	−0.07	−0.28	−0.15
Gizzard (g)																	
Empty						0.88 ****	0.41 *	0.13	0.13	0.42 *	0.36 *	0.39 *	0.05	0.40 *	−0.16	0.31	0.10
Full							0.57 ***	0.47 **	0.26	0.38 *	0.15	0.26	−0.05	0.35 *	−0.24	0.19	0.08
Fraction 1 (500–1000 µm)								−0.01	0.11	0.13	−0.18	0.17	−0.04	0.19	0.05	0.11	−0.03
Fraction 2 (1000–1500 µm)									0.15	0.21	−0.15	−0.10	−0.07	−0.10	−0.31	−0.12	0.12
Fraction 3 (1500–2000 µm)										−0.04	−0.06	0.15	−0.18	0.37 *	0.02	0.09	−0.15
Digestive tract mes. (cm)																	
Small intestine length (duodenum + jejunum + ileum)											0.48 **	0.48 **	0.26	−0.16	0.01	0.09	0.20
Caeca length												0.16	0.07	0.05	−0.26	0.06	0.19
Colon length													0.08	0.30	0.18	0.27	−0.03
Small intestine hist. mes.																	
Villus height (µm)														−0.08	0.04	0.74 ****	0.76 ****
Villus width (µm)															−0.24	0.61 ***	0.07
Crypt depth (µm)																−0.12	0.59 ***
Villus area (µm^2^)																	0.66 ****

* *p* < 0.05; ** *p* <0.01; ***p < 0.001; **** *p* < 0.0001.

**Table 4:** Spearman correlations between gastrointestinal tract morphometrics and content of Sasso chickens.

	Crop (g)				Gizzard (Muscular Stomach) (g)					Digestive Tract Measurements (cm)			Small Intestine Histological Measurements				
	Full	Feed Content	Pasture Matter	Feather	Empty	Full	Fraction 1 (600–1000 µm)	Fraction 2 (1000–1400 µm)	Fraction 3 (1400–1800 µm)	Small Intestine Length (Duodenum + Jejunum + Ileum)	Caeca Length	Colon Length	Villus Height (µm)	Villus Width (µm)	Crypt Depth (µm)	Villus Area (µm^2^)	Villus Height/Crypt Depth
Crop (g)																	
Empty	0.63 ***	0.54 **	0.34	0.10	0.24	0.22	0.02	0.08	0.24	0.26	0.23	0.23	0.33	−0.02	0.18	0.23	0.13
Full		0.99 **	0.51 **	0.11	0.26	0.35	0.25	0.18	0.37	0.11	0.16	−0.10	0.18	0.26	0.16	0.33	0.01
Feed content			0.50 **	0.10	0.26	0.35	0.26	0.17	0.37	0.06	0.13	−0.15	0.16	0.29	0.15	0.33	0.00
Pasture matter				−0.17	0.13	0.13	0.01	0.05	0.36	0.41 **	0.23	0.15	0.13	−0.20	−0.36	−0.04	0.38 *
Feather					−0.28	−0.20	−0.06	−0.13	−0.15	−0.14	−0.31	0.03	−0.15	0.13	0.20	−0.03	−0.19
Gizzard (g)																	
Empty						0.83 ****	0.39 *	0.38 *	0.46 *	0.05	0.07	0.07	0.10	0.04	−0.05	0.12	0.07
Full							0.81 ****	0.69 ****	0.53 **	−0.02	0.03	−0.12	0.09	0.21	−0.03	0.23	0.05
Fraction 1 (500–1000 µm)								0.65 ****	0.29	−0.10	0.01	−0.30	0.00	0.29	0.01	0.21	−0.03
Fraction 2 (1000–1500 µm)									0.01 **	−0.25	0.03	−0.04	0.23	0.04	0.04	0.21	0.12
Fraction 3 (1500–2000 µm)										0.00	−0.13	−0.11	0.27	0.34	−0.07	0.42 *	0.24
Digestive tract mes. (cm)																	
Small intestine length (duodenum + jejunum + ileum)											0.64 ***	0.52 **	0.06	−0.03	−0.13	0.04	0.13
Caeca length												0.47 *	0.36	−0.19	−0.04	0.14	0.28
Colon length													0.24	−0.40 *	−0.15	−0.11	0.32
Small intestine hist. mes.																	
Villus height (µm)														0.00	0.28	0.71 ****	0.61 ***
Villus width (µm)															0.47	0.70 ****	0.39 *
Crypt depth (µm)																0.56 **	−0.58 ***
Villus area (µm^2^)																	0.13

* *p* < 0.05; ** *p* < 0.01; *** *p* < 0.001; **** *p* < 0.0001.
